# Effects of parents training on parents’ knowledge and attitudes about adolescent sexuality in Accra Metropolis, Ghana

**DOI:** 10.1186/s12978-017-0363-9

**Published:** 2017-08-24

**Authors:** Elizabeth Aku Baku, Isaac Agbemafle, Richard M. K. Adanu

**Affiliations:** 1 School of Nursing and Midwifery, University of Health and Allied Sciences, PMB 31, Ho, Volta Region Ghana; 2 Department of Family and Community Health, School of Public Health, University of Health and Allied Sciences, Ho, Volta Region Ghana; 3 Department of Population, Family and Reproductive Health, Office of the Dean, School of Public Health, University of Ghana, Legon, Greater-Accra Region Ghana

**Keywords:** Adolescents, Parents, Sexuality, Reproductive health, Knowledge, Attitudes, Accra

## Abstract

**Background:**

Attitudes of parents about discussing sexuality issues with adolescents may or may not be influenced by their level of knowledge on such issues. This study seeks to examine parents’ training and its effects on parent knowledge and attitudes about sexuality in Accra Metropolis, Ghana.

**Methods:**

This was an intervention study consisting of 145 parents who were recruited through their wards from 12 public junior high schools in Accra Metropolis. Parents were randomized equally into intervention and control groups and the intervention group received a 4 weeks training on adolescent sexuality topics. At pre-intervention and 3 months after parent training, parents answered questions on knowledge and attitudes about adolescent sexuality. Changes in baseline and follow-up within and between groups were compared using the difference- in-difference model and logistic regression.

**Results:**

The ages of the parents ranged from 26 to 63 years and 44.1% of them completed middle school. There were 69.9% and 59.7% mothers in the intervention and control groups respectively. At pre-intervention, 21.9% of parents in the intervention group had very good knowledge but this increased significantly to 60% three months after the training. Knowledge about sexuality increased to a lesser degree from 18.1% to 34.7% in the control group. Parents’ positive attitudes towards adolescents’ sexuality increased by 50% in the intervention group compared to 20% in the control group. There were significant differences in knowledge on adolescent sexuality as parents in the intervention group had a greater positive effect than parents in the control group (28.7%, *p*-value = <0.001). Regarding attitudes of parents towards allowing adolescents to use family planning services (FPS), there was a greater positive effect on parents in the intervention group compared to those in the control group (37.4%; *p*- value  = <0.001). Being part of the intervention group increased the odds of parent knowledge on adolescent sexuality by 16-fold (*p*-value = <0.001), whilst being in the intervention group increased the likelihood of parents’ attitudes towards allowing adolescents to use FPS by four fold (*p*-value = 0.039).

**Conclusion:**

Training parents for a relatively short period of time can positively impact parents’ knowledge and attitudes about adolescent sexuality. This may have beneficial effects on adolescent reproductive health.

## Plain language summary

Knowledge is important because it provides the foundation for human behaviour and attitudes. This study assessed the effect of parent training on knowledge and attitudes about adolescent sexuality in Accra Metropolis, Ghana. Parents were put into two groups namely the control and intervention groups. The intervention group received a one month training on adolescent sexuality and the control group received no training. The parents answered the same questions before (pre-intervention) and three months after the training (post-intervention) to assess the effect of the training.

At pre-intervention, there was no difference in knowledge and attitudes between the parents in both groups. At post-intervention, there were major differences in knowledge on adolescent sexuality as parents in the intervention group had a greater positive effect than parents in the control group. Regarding attitudes of parents towards allowing adolescents to use family planning services, there was a greater positive effect on parents in the intervention group as compared to those in the control group. Being part of the intervention group increased the likelihood of parents’ knowledge about adolescent sexuality by 16-times. Also, taking part in the training programme increased the likelihood of parents’ attitudes towards allowing adolescents to use family planning services by four fold.

In conclusion, training parents on sexuality increased parents’ knowledge on adolescent sexuality and positive attitudes towards allowing adolescents to use family planning services and this may have beneficial effects on adolescent reproductive health.

## Background

Sexuality is part of human life and development. It develops during childhood and accelerates through adolescence. During adolescence young people strengthen their gender identities and begin clarifying their sexual orientations and identities as they experience more adult-like erotic feelings and experiment further with sexual development. A study conducted among Canadian East Asian men reported that, more than half of 17 year olds have had sexual intercourse and by the end of adolescence, the majority of them have had sexual intercourse [1]. In Ghana, one in three adolescents in slums have ever had sexual intercourse and the proportion is heavily skewed towards those who are not in school [2]. Also, one-third of the adolescents who had ever had sexual intercourse reported that their first sexual intercourse was unplanned [2]. Although data from the 2014 Ghana Demographic and Health Survey is censored, it was evident that a greater proportion of adolescents in rural areas had ever had sexual intercourse as compared to the adolescents living in urban areas [3]. Thus, adolescent sexual and reproductive health issues are prevalent in Ghana and may often go unreported by adolescents. This poses several challenges to parents, teachers, and healthcare providers and to the adolescents themselves**.** This has fuelled controversies regarding how best to educate adolescents about sex in order to prevent unplanned pregnancies and sexually transmitted infections (STIs).

According to Arnett [4] adolescence is a period of healthy life including sexual life, but many adolescents are less informed about their sexual and reproductive health by their parents. Several studies indicate that sex education should not be left to parents alone, but should also involve schools, communities and the media [5–8]. A study of parents regarding sex education of adolescents in New Brunswick, Canada, found that 95% agreed that the responsibility for sex education should be shared by the school and the home [5]. The researchers pointed out that school and community values may differ and that the best sexual health should start at home. The study further noted that adolescents may experience disapproval or even unfriendliness and bad approaches from adults in their attempt to obtain the reproductive information they need [5]. Sedge et al. [9] noted that while the value of sex education is acknowledged, the intervention is opposed in many African societies including Ghana, particularly due to the argument that early exposure to knowledge about sexuality and reproductive health could do more harm to adolescents than helping them to overcome problems they faced in growing up. This indicates that socio-cultural factors must be acknowledged in adolescent sexual and reproductive health education.

Ankomah [10] established that sex education lessons were mainly provided during initiation rites and ceremonies to provide girls with information mainly on how to ‘sleep’ with their husbands, menstrual taboos, how to recognize pregnancy and maintain personal hygiene by recognized older women who serve as custodians of instructions on motherhood. This study was published 16 years ago and this may have changed since then. Sex education, which is generally a school-based programme taught by teachers, has been demonstrated to be useful to adolescents and has helped to delay sexual activity among adolescents [11]. Yet, many adolescents rely on their peers for information about sex that may be inaccurate. Adolescents need the facts and correct information on their sexual and reproductive health. Parents as primary educators could be the key people in decreasing adolescents’ sexual-risk taking by parent-adolescent communication about sex but many parents lack the skills [6]. Thus, parents must be empowered with the necessary skills to talk to their children about sexual and reproductive health issues. This study seeks to assess the effect of training of parents on adolescent sexual and reproductive health topics on their knowledge and attitudes about adolescent sexuality topics in Accra, Ghana.

## Methods

### Study design and setting

This was a randomized controlled field trial designed to assess the effect of parents training on knowledge and attitudes about adolescent sexuality. The study was conducted with parents in 12 public Junior High Schools (JHS) in the Osu Klottey and Ablekuma South sub-metropolises, Accra Metropolis, Ghana. The Accra Metropolis has 11 sub-metropolises, and the study was conducted in these two sub-metropolises because they were geographically far apart. The Osu Klottey Sub-metropolis is one of the oldest Ga communities along the Coast in Accra. The inhabitants are mainly fishermen and fishmongers and there are 19 public JHS in Osu Klottey. Ablekuma South is a newly-created sub-metropolis and it is cosmopolitan in nature with 37 public JHS.

### Sampling and sample size

A multi-stage random sampling procedure was used. The first stage involved the random selection of the two sub-metropolises from Accra Metropolis by simple balloting. After that all the public JHS in each sub-metropolis were listed and six JHS were selected randomly in each sub-Metropolis by hand picking after the folded ballot papers had being placed in a box and mixed thoroughly. The schools were randomized into intervention and control groups at the sub-metropolis level: Osu Klottey as the intervention and Ablekuma South as the control sub-metropolis. Assuming 42% [7] of parents communicated with adolescent about sexuality, alpha level of 0.05 and 80% power was used to obtain a sample size of 69 parents in each group in order to detect an effect size of 60% at 5% level of significance.

**Fig. 1:**
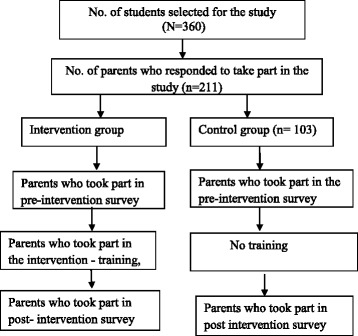
Flow of participation of parents throughout the study

Thirty adolescent (12 to 17 years) students from each of the 12 schools consisting of ten students each from forms 1, 2, and 3 were selected by simple balloting. The 180 students selected from each of the two sub-metropolis were given invitation letters and consent forms to send to their parents inviting them to participate in the study. A total of 211 parents, comprising of 108 from the intervention and 103 from the control group, indicated their willingness to be part of the study. Assessment was conducted at pre-intervention and 3 months after the intervention. The flow diagram for the study is as shown in Fig. 1. The study was approved by the Noguchi Memorial Institution for Medical Research Review Board (IRB no. 097/11–12).

### Data collection

#### Pre-intervention survey

Information on parents’ socio-demographic characteristics, their knowledge about sexuality and attitudes towards adolescent sexuality were obtained at baseline. Questionnaires were administered through one-on-one interview. The questionnaire consisted of 25 questions on sexuality topics. The questionnaire was used to assess parents’ perceptions and knowledge about adolescent sexuality. The parents were asked to rate their knowledge about the 25 questions on the sexuality topics. The parents were asked to “circle the answer which agrees with how you rate your knowledge and understanding of the following sexual terms and statements” with the responses measured on a 4-point likert scale “1 = no knowledge”, “2 = little knowledge” “3 = good knowledge” and “4 = very good knowledge”. The 25 questions on sexuality topics were grouped under five topics: biological development, sexual risk protection, contraceptive use, risky sexual behaviours, and experiential sex.

Biological development was assessed using the following six questions: how girls’ bodies change physically as they grow up, how boys’ bodies change physically as they grow up, menstruation, wet dreams among boys (as a sign of maturity to produce semen and sperm), how girls get pregnant and have babies and masturbation. Under risky sexual behaviour topics six questions were asked: consequences of having premarital sex, consequences of getting pregnant/getting somebody pregnant, consequences of abortion if one gets pregnant/gets someone pregnant, pressure from friends to have sex, symptoms of sexually transmitted diseases, and effects of substance (alcohol, smoking) use on sex.

Sexual risk protection was assessed using nine questions: how to prevent pregnancy, abstinence from sex until marriage, how to decide whether or not to have sex, how to overcome pressure from friends to have sex, reason why your child should not have sex, how the adolescent can say ‘no’ if somebody wants to have sex with your child and s/he does not want to, what your child will do when a partner does not want to use condom and s/he wants to have sex with her/him, how people can prevent getting sexually transmitted diseases, how to prevent getting HIV/AIDS. Parents’ knowledge on contraceptive use was assessed using the following two questions: uses of condom/how to use condom and use of other methods of contraception to prevent pregnancy. For the last category, two questions were used to measure parents’ knowledge on experiential sexual topics: having sexual feelings and having heard about issues of homosexuality (gays/lesbians).

Lastly, two questions were used to evaluate parents’ attitudes towards allowing adolescents to use family planning services (FPS): would you allow your son to get reproductive health services e.g. family planning? would you allow your daughter to get reproductive services e.g. family planning? Response to these questions were scored on a 3-point likert scale: 1 = disallow, 2 = neutral and 3 = allow. Other attitudes-related questions included: “would you approve of discussing sexual topics with your child/children” and “would you allow your child to use condoms if s/he is engaging in sex” were asked and scored as reported for the two-item attitudes questions. Questionnaire was pre-tested before use and all completed questionnaires retrieved were cross-checked for consistency and all necessary corrections made. The research assistants and workshop facilitators were trained on data collection procedures and discussion of adolescent sexuality topics respectively.

### Intervention/Training programme

The parents’ health education training programme (intervention) was adapted from ‘Talking Parents, Healthy Teens’ to train parents to communicate effectively with their children about sexuality [8]. The training focused on building a relationship with your child, child development, talking about sex, parent helping child to make decisions about sex, assertiveness skills, abstinence and contraceptive use. The intervention group was divided into two groups and were taught skills to communicate with their children. The programme was held on Saturdays for 2 h per session for a period of four weeks for each group in the intervention arm. The two groups of parents in the intervention were trained simultaneously. Each group consisted of about 35 parents. Each parent undertook 8 h of structured training.

For week one training, the parents were given an overview of the training programme and reason for organizing it. This first session also focused on positive parent-child relationship: the importance of talking to children about sex; establishing a quality parent-child relationship; identifying and reinforcing children’s strengths; spending time with children and supervising children. Lessons for week two focused on adolescent physical, social, emotional and cognitive development. Parents were taught that some adolescent behaviour which may seem bizarre and frustrating may be part of adolescent normal development. They were taught how physical changes may affect the way adolescents feel about themselves and that adolescent’s sexual and romantic feelings were developing.

For week three, parents considered the adolescent perspectives on sexual matters, and anticipated potential adolescent responses. This session addressed assertiveness skills for adolescents who want to remain abstinent from sexual activity in a particular situation, as well as methods of preventing STIs or unintended pregnancies among adolescents who engaged in sexual activity. The last training session involved strategies for negotiating conflicts. Parents were taught adolescents’ assertive skills that they could use if they decide to talk about sex and contraception. Also, parents were taught to help the adolescent develop decision –making skills using the **S.T.O.P**. steps: **S**tate the decision, **T**alk about feelings and needs, brainstorm and discuss **O**ptions and **P**ick the best option and later evaluate. The sessions were very interactive and included role-plays and demonstrations. Parents in the control group did not receive any training and information about adolescent sexuality topics.

### Post-intervention survey

The post intervention survey was conducted 3 months after the training. This was used to assess the effects of the training on parents’ knowledge and attitudes about adolescent sexuality. The same questionnaire used in the pre-intervention survey was used to assess knowledge and attitudes three months after the training for the parents in both the intervention and control groups who remained in the study after their initial enrolment. Parents completed the post-intervention survey at home and sent the completed questionnaires through their children to their schools. The signed completed parent’s questionnaire was delivered by child in a sealed envelope to ensure that child did not temper with the questionnaire. Parents were called over the phone to confirm the day of submission of their completed questionnaire and for confirmation of date the questionnaire was completed and/or delivered to child to be brought to school. Any envelope that was tempered with by either bearer - child or teacher – would be rejected. There was no evidence that envelopes received from the children or teacher were tempered with.

### Statistical analysis

Biological development and risky sexual behaviour were categorized into quartiles based on a minimum score of six (for a parent who answered “1 = no knowledge” for the six questions) and a maximum score of 24 (for a parent who answered “4 = very good knowledge” for the six questions). The scores were categorized as follows: "6–10 = no knowledge", "11–15 = little knowledge", "16–19 = good knowledge" and "20–24 = very good knowledge". A total score of 36 was obtained for the nine questions on sexual risk protection if parents answered “4 = very good knowledge”. The total score was then grouped into four levels of “9–15 = no knowledge”, “16–22 = little knowledge”, “23–29 = good knowledge” and “30–36 = very good knowledge”. A minimum score of two was obtained if a parent responded “1 = no knowledge” and a maximum score of 8 was obtained if a parent responded “4 = very good knowledge” to each of the two-item questions for contraceptive use and experiential sexual topics. The total score was grouped into four levels: "2–3 = no knowledge", "4–5 little knowledge", "6–7 = good knowledge" and "8 = very good knowledge".

The total score for the 25 questions under parents’ knowledge about sexuality topics was summed up and a score of 25 and 100 was obtained for parents who answered 1 = no knowledge and 4 = very good knowledge respectively. The scores were categorized into three levels of "25–50 = little knowledge", "51–75 = good knowledge" and "76–100 = very good knowledge". The higher the score a parent had, the higher his/her knowledge about adolescent sexuality. The scores of the two-item questions used to assess parents’ attitudes were summed and a minimum score of two and a maximum score of six was attained if a parent answered “1 = disallow” and “3 = allow” to all the two questions. The total score was grouped into three levels: “2–3 = disallow”, “4–5 = neutral” and “6 = allow”. Higher scores indicated parents’ positive attitudes towards allowing adolescents’ to use FPS.

The data were entered, recoded and analysed using SPSS version 20. For the purposes of running a logistic regression, all outcome variables (knowledge and attitudes) were recoded as binary data. Specifically, for all the 25 questions on parents’ knowledge about sexuality topics, a minimum and maximum score of 25 and 100 was obtained for overall parent’s knowledge; these scores were then categorized into two groups of "25–63 for little knowledge" and "64–100 for good knowledge". Codes of 0 and 1 were assigned to little knowledge and good knowledge respectively for analysis purposes. Parents’ attitudes about adolescent use of FPS was regrouped as follows: “0 = 1–4 disallow”, “1 = 5–6 allow”.

The descriptive data were summarized into frequency tables. Pearson’s Chi-square test was used to find the relationship between the intervention and control groups. Based on the binary nature of the outcome variables, a binary logistic regression was used to investigate the influence of the education programme and the resulting estimates were adjusted for age, sex and marital status of parents. The difference-in-difference (DID) model was used to measure the effect of the training programme between the intervention and control group. DID is the difference in the average outcome in the intervention group before and after the intervention and the difference in average outcome in the control group before and after the intervention. For each outcome under study, DID was estimated by calculating difference between proportion of participants at baseline and end of study for the intervention and control groups separately, using the two-sample test for binomial proportions for categorical data [12]. The result of the difference obtained for the control group was then subtracted from the result of the difference obtained for the intervention group. The differences in the different means was then compared and a *p*-value less than 0.05 was considered significant.

## Results

### Background characteristics

One hundred and forty-five parents were recruited into the study. The two groups consisted of 64.8% mothers and 35.2% fathers as shown in Table 1. Specifically, 69.9% of parents in the intervention group were mothers while the control group consisted of 59.7% of mothers. The ages of the parents ranged from 26 to 63 years, with the majority of them in the 45–54 years age group. About 72.2% of parents in the control group were married as compared to 53.4% in the intervention group. Almost half (44.1%) of the parents had middle school level of education. More parents in the control group had higher education as compared to the intervention group (30.6% vs. 21.9%; *p*-value = 0.38, Table 1). Most (93.8%) of the parents were Christians and the remaining were Moslems. With regard to occupation, the majority (60.7%) of the parents were either traders or artisans, 24.1% worked in the formal sector and 15.2% in the private sector (Table 1).

**Table 1:** Socio-demographic characteristics of the parents

Characteristics	Total [*N* = 145; n (%)]	Intervention [*n* = 73; n (%)]	Control [*n* = 72; n (%)]	*p*-value
Sex				
Male	51 (35.2)	22 (30.1)	29 (40.3)	0.201
Female	94 (64.8)	51 (69.9)	43 (59.7)	
Age group				
25–34	24 (16.6)	17 (23.3)	7 (9.7)	0.078
35–44	49 (33.8)	22 (30.1)	27 (37.5)	
45–54	56 (38.6)	24 (32.9)	32 (44.4)	
≥ 55	16 (11.0)	10 (13.7)	6 (8.3)	
Marital status				
Married/Living together	91 (62.8)	39 (53.4)	52 (72.2)	0.135
Divorced/Separated	26 (17.9)	16 (21.9)	10 (13.9)	
Widowed	12 (8.3)	8 (11.0)	4 (5.6)	
Never married	16 (11.0)	10 (13.7)	6 (8.3)	
Education level				
Middle school/JSS/JHS	64 (44.1)	36 (49.3)	28 (38.9)	0.375
SSS/SHS/Technical/Vocational	43 (29.7)	21 (28.8)	22 (30.6)	
Tertiary/Training college	38 (26.2)	16 (21.9)	22 (30.6)	
Ethnicity				
Akans	50 (34.5)	26 (35.6)	24 (33.3)	0.609
Gas/Dangmes	61 (42.1)	27 (37.0)	34 (47.2)	
Ewes	18 (12.4)	10 (13.7)	8 (11.1)	
Northerners	15 (10.3)	10 (13.7)	6 (8.3)	
Religious affiliation				
Catholic	12 (8.3)	6 (8.2)	6 (8.3)	0.082
Anglican	5 (3.4)	4 (5.5)	1 (1.4)	
Presbyterian	21 (14.5)	11 (15.1)	10 (13.9)	
Methodist	19 (13.1)	9 (12.3)	10 (13.9)	
Pentecostal/Charismatic	79 (54.5)	35 (48.0)	44 (61.1)	
Moslem	9 (6.2)	8 (11.0)	1 (1.4)	
Occupation				
Trader	64 (44.1)	36 (49.3)	28 (38.9)	0.05
Artisan	24 (16.6)	16 (21.9)	8 (11.1)	
Public servants	35 (24.1)	12 (16.4)	23 (31.9)	
Private sector	22 (15.2)	9 (12.3)	13 (18.1)	
Income level (GHC)				
0–199	28 (19.3)	18 (24.7)	10 (13.9)	0.252
200–599	46 (31.7)	23 (31.5)	23 (31.9)	
600–999	35 (24.1)	19 (26.0)	16 (22.2)	
1000–1599	26 (17.9)	9 (12.3)	17 (23.6)	
≥1600	10 (6.9)	4 (5.5)	6 (8.3)	

*JSS* Junior Secondary School, *JHS* Junior High School, *SSS* Senior Secondary School, *SHS* Senior High School

### Parents’ knowledge about adolescents’ sexuality topics

At pre-intervention, all parents had some knowledge about adolescent sexuality topics. Parents’ exhibited very high level of knowledge on biological development topics whilst the level of knowledge on contraceptive use was low. At pre-intervention, both the intervention and control groups had almost equal proportions of parents with good knowledge about biological development topics. However, the proportion of parents in the intervention group with very good knowledge about biological development topics was slightly higher than parents with good knowledge (35.6% vs. 29.2%, *p*-value = 0.589; Table 2). On sexual risk protection topics, a smaller proportion of parents in the intervention group had good knowledge or very good knowledge as compared to the control group (75.3% vs. 82.0%). At pre-intervention, there were no significant differences in parents knowledge about adolescent sexuality topics.

**Table 2:** Parents’ perceived knowledge about adolescent sexuality topics

Topics	Pre-intervention (*N* = 145)			Post intervention (*N* = 142)		
	Intervention (*n* = 73)	Contro1 (*n* = 72)	*p*-value	Intervention (*n* = 70)	Control (*n* = 72)	*p*-value
Biological development topics						
Little knowledge	13 (17.8)	17 (23.6)	0.589	0 (0.0)	10 (13.9)	0.001*
Good knowledge	34 (46.6)	34 (47.2)		32 (45.7)	36 (50.0)	
Very good knowledge	26 (35.6)	21 (29.2)		38 (54.3)	26 (36.1)	
Sexual risk protection topics						
No knowledge	1 (1.4)	1 (1.4)	0.358	0 (0.0)	0 (0.0)	0.002*
Little knowledge	17 (23.3)	12 (16.7)		0 (0.0)	10 (13.9)	
Good knowledge	20 (27.4)	29 (40.3)		19 (27.1)	22 (30.6)	
Very good knowledge	35 (47.9)	30 (41.7)		51 (72.9)	40 (55.6)	
Contraceptive use topics						
No knowledge	27 (37.0)	17 (23.6)	0.184	1 (1.4)	15 (20.8)	0.002*
Little knowledge	14 (19.2)	22 (30.6)		22 (31.4)	18 (25.0)	
Good knowledge	13 (17.8)	17 (23.6)		30 (42.9)	22 (30.6)	
Very good knowledge	19 (26.0)	16 (22.2)		17 (23.3)	17 (23.6)	
Risky sexual behaviours topics						
No knowledge	6 (8.2)	2 (2.8)	0.275	0 (0.0)	0 (0.0)	0.002*
Little knowledge	16 (21.9)	17 (23.6)		0 (0.0)	8 (11.1)	
Good knowledge	23 (31.5)	31 (43.1)		30 (42.9)	38 (52.8)	
Very good knowledge	28 (38.4)	22 (30.6)		40 (57.1)	26 (36.1)	
Experiential sexual topics						
No knowledge	13 (17.8)	13 (18.1)	0.221	1 (1.4)	7 (9.7)	0.009*
Little knowledge	18 (24.7)	27 (37.5)		10 (14.3)	22 (30.6)	
Good knowledge	29 (39.7)	18 (25.0)		35 (50.0)	25 (34.7)	
Very good knowledge	13 (17.8)	14 (19.4)		24 (34.3)	18 (25.0)	

*Significant at *p*-value <0.05

After the intervention or training programme, a higher proportion of parents in the intervention group either had good knowledge or very good knowledge about adolescents’ biological development topics as compared to the parents in the control group (100% vs. 86.1%, *p* = 0.001; Table 2). A similar and statistically significant trend was observed for sexual risk protection, contraceptive use, risky sexual behaviour and experiential sexual topics between the intervention and control groups before and after the training programme. Increase in knowledge about biological development, sexual risk protection, risky sexual behaviour and experiential sexual topics were much higher than for contraceptive use in both the intervention and control groups. For example, more than half (56.2%) of parents in the intervention group had little or no knowledge about contraceptive use topics but after the intervention, this dropped to 32.8% as shown in Table 2. Also, about 18.0% of parents in the intervention group had no knowledge about experiential sexual topics but after the intervention, this reduced to 1.4% (*p*-value = 0.009; Table 2).

The study also looked at the knowledge of mothers and fathers separately using the same categories of adolescent sexuality topics as shown in Table 3. There were significant differences in mothers’ knowledge from pre-intervention to post-intervention within the groups. Also, the proportion of fathers in the intervention with very good knowledge about adolescent sexuality topics was significantly higher than the proportion at pre-intervention. There were differences in knowledge about adolescent sexuality topics for mothers and fathers in each sex category at post-intervention, but this difference was not observed at pre-intervention. At pre-intervention, there were no difference in knowledge among the mothers nor the fathers. At post-intervention, there were differences in knowledge either among the mothers or the fathers and this was significant within each sex category as shown in Table 3.

**Table 3:** Mothers’ and Fathers’ perceived knowledge about adolescent sexuality topics

Topics	Mothers’						Fathers’					
	Pre- intervention (*N* = 94)			Post intervention (*N* = 92)			Pre- intervention (*N* = 51)			Post intervention (*N* = 51)		
	Intervention (*n* = 51)	Control (*n* = 43)	*p*- value	Intervention (*n* = 48)	Control (*n* = 43)	*p*-value	Intervention (*n* = 22)	Control (*n* = 29)	*p*- value	Intervention (*n* = 22)	Control (*n* = 29)	*p*-value
Biological development topics												
Little knowledge	9(17.6)	7(16.3)	0.839	0(0.0)	7(15.9)	0.004	5(22.7)	9(31.0)	0.747	0(0.0)	6(20.7)	0.016
Good knowledge	23(45.1)	22(51.2)		20(41.7)	20(46.5)		10(45.5)	13(44.8)		7(31.8)	13(44.8)	
Very good knowledge	19(37.3)	14(32.6)		28(58.3)	16(37.2)		7(31.8)	7(24.1)		15(68.2)	10(34.5)	
Sexual risk protection topics												
No knowledge	0(0.0)	1(2.3)	0.737	0.0	0.0	0.005	1(4.5)	0(0.0)	0.161	0(0.0)	0(0.0)	0.001
Little knowledge	10(19.6)	6(14.0)		0(0.0)	8(18.6)		6(27.3)	7(24.1)		0(0.0)	10(34.5)	
Good knowledge	15(29.4)	14(32.6)		11(22.9)	8(18.6)		5(22.7)	14(48.3)		4(18.2)	8(27.6)	
Very good knowledge	26(51.0)	22(51.2)		37(77.1)	27(62.8)		10(45.5)	8(27.6)		18(81.8)	11(37.9)	
Contraceptive use topics												
No knowledge	18(35.3)	12(27.9)	0.318	1(2.1)	8(18.6)	0.015	8(36.4)	5(17.2)	0.374	1(4.5)	7(24.1)	0.026
Little knowledge	10(19.6)	11(25.6)		20(41.7)	9(20.9)		5(22.7)	11(37.9)		2(9.1)	9(31.0)	
Good knowledge	8(15.7)	12(27.9)		19(39.6)	15(34.9)		5(22.7)	5(17.5)		10(45.5)	7(24.1)	
Very good knowledge	15(29.4)	8(18.6)		8(16.7)	11(25.6)		4(18.2)	8(27.6)		9(40.9)	6(20.7)	
Risky sexual behaviours topics												
No knowledge	18(35.3)	12(27.9)	0.318	0(0.0)	0(0.0)	0.008	2(9.1)	0(0.0)	0.374	0(0.0)	0(0.0)	0.011
Little knowledge	10(19.6)	11(25.6)		0(0.0)	7(15.9)		6(27.3)	11(37.9)		0(0.0)	7(24.1)	
Good knowledge	8(15.7)	12(27.9)		19(39.6)	17(39.5)		7(31.8)	11(37.9)		11(50.0)	16(55.2)	
Very good knowledge	15(29.4)	8(18.6)		29(60.4)	19(44.2)		7(31.8)	7(24.1)		11(50.0)	6(20.7)	
Experiencing sex topics												
No knowledge	3(5.9)	2(4.7)	0.359	1(2.1)	7(16.3)	0.016	3(13.6)	6(20.7)	0.159	1(4.5)	8(27.6)	0.006
Little knowledge	12(23.5)	6(14.0)		7(14.6)	13(30.2)		5(22.7)	13(44.8)		3(13.6)	11(37.9)	
Good knowledge	15(29.4)	20(46.5)		21(43.8)	13(30.2)		11(50.0)	6(20.7)		9(40.9)	6(20.7)	
Very good knowledge	21(41.2)	15(34.9)		19(39.6)	10(23.3)		3(13.6)	4(13.8)		9(40.9)	4(13.8)	

**Fig. 2:**
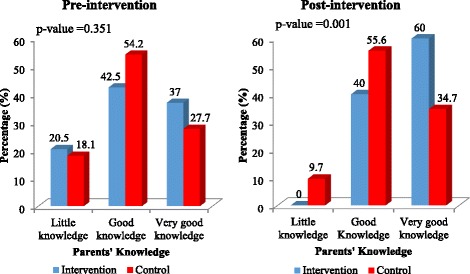
Parents’ knowledge about adolescent sexuality topics

At pre-intervention, 20.5% of the parents in the intervention group had little knowledge about adolescent sexuality topics as compared to 18.1% in the control group. After the training, none of the parents in the intervention group had little knowledge about adolescent sexuality topics as compared to 9.1% in the control group as shown in Fig. 2. There were no significant differences in very good knowledge levels of parents in both groups at pre-intervention (37.0% vs. 27.7%, *p*-value = 0.351; Fig. 2). After the intervention, significantly more parents in the intervention group displayed very good knowledge levels (60.0% vs. 34.7%, *p*-value = 0.001; Fig. 2).

### Parents’ attitudes towards allowing adolescents to use family planning services

**Fig. 3:**
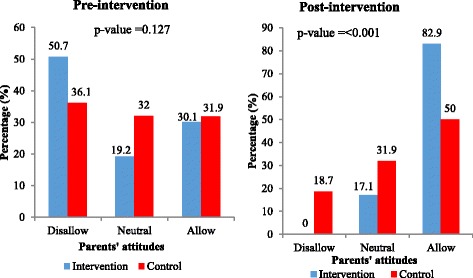
Parents’ attitudes of approval towards adolescents’ use of family planning services

**Fig. 4:**
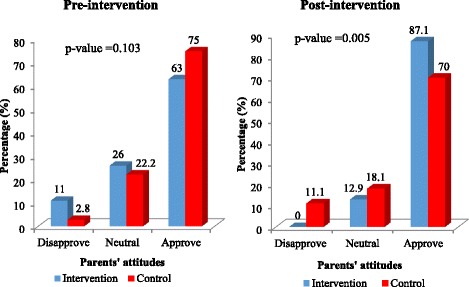
Parents’ attitudes towards discussing sexual topics with adolescents

**Fig. 5:**
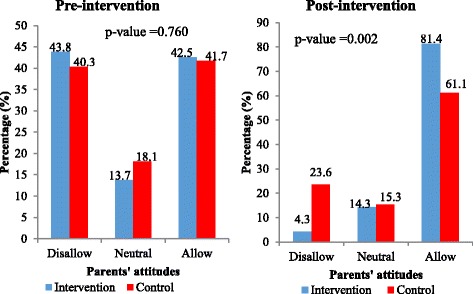
Parents’ attitudes of approval towards sexually active adolescents’ use of condoms

At pre-intervention, about half (50.7%) of parents in the intervention group and more than one-third (36.1%) in the control group would disallow their adolescents to use FPS. At post-intervention, none of the parents in the intervention group would disallow their adolescents to use FPS whilst about 18% of parents in the control group would still disallow their adolescents to use FPS as shown in Fig. 3. After the training, 82.9% as compared to 30.1% at pre-intervention of the parents in the intervention group were willing to allow their adolescent to use FPS whilst the control group increased to 50.0% over the baseline proportion of 31.9% (Fig. 3). Thus parents’ attitudes toward allowing adolescents to use FPS increased sharply after the training about adolescent sexuality topics and this was statistically significant between the two groups. As shown in Fig. 4, only 11.0% of the parents in the intervention and 2.8% in the control groups specified that they would disapprove of discussing sexuality topics with their adolescents before the training. There was an increase among parents in the intervention group who reported that they would approve of discussing sexuality topics with their adolescents from 63.0% to 87.1% as compared to a decrease from 75.0% to 70.8% among the parents in the control group (*p*-value = 0.005; Fig. 4). Before the intervention about 43.8% of the parents in the intervention group and 40.3% in the control group stated that they would not allow their sexually active adolescents to use condoms. After the intervention, parents who indicated that they would allow their sexually active adolescents to use condoms rose from 42.5% to 81.4% among the intervention group as compared to an increase from 41.7% to 61.1% for parents in the control group (*p*-value = 0.002; Fig. 5).

### Changes in knowledge and attitudes after parents training

The difference-in-difference (DID) obtained for parents’ knowledge about adolescent sexuality topics showed that the intervention had a greater positive effect on parents in the intervention group than the parents in the control group (28.7%, *p* = <0.001; Table 4). Regarding attitudes of parents towards allowing adolescents to use FPS, there was a greater positive effect on parents in the intervention group as compared to those in the control group (37.4%; *p*-value = <0.001; Table 4).

**Table 4:** Effects of parents training on parents' knowledge and attitudes on  adolescent sexuality topics

	Pre-intervention^a^	Post-intervention^b^	Difference^c^	Difference in differences
Knowledge of parents on sexual reproductive health of adolescents				
Intervention [70]	0.6286 (0.058)	0.9571 (0.024)	0.3285 (0.069)	0.287 (0.065)
Control [72]	0.5278 (0.0592)	0.5694 (0.059)	0.0416 (0.083)	*p* < 0.001
Attitudes of parents towards use of family planning services by adolescents				
Intervention [70]	0.4143 (0.059)	0.8571(0.042)	0.4428 (0.081)	0.374 (0.073)
Control [72]	0.5556 (0.0590)	0.6250 (0.058)	0.0694 (0.082)	*p* < 0.001

^a^Proportion of participants at baseline

^b^Proportion of participants at end line

^c^Difference (absolute) in pre and post intervention proportions of participants who were part of either the treated or control group obtained from two-sample test for binomial proportions (normal theory test)

In this study, the odds or likelihood of an increase in knowledge and attitudes at the end of the training was compared for the intervention and control groups. The odds that a parent gained knowledge about adolescent sexuality after participating in the intervention, as opposed to the control group, when all other factors were held constant was 16.2 (*p*-value = <0.001; Table 5). This means that being part of the intervention group increases the odds of parents knowledge about adolescent sexuality topics by 16-fold. Also, the odds of attitudes of parents toward allowing adolescents to use FPS after the training in the intervention group compared to the control group when all other factors were held constant is 3.72 (*p*-value = 0.039; Table 5). This shows that being part of the intervention group increases the likelihood of parents’ attitudes towards allowing adolescents to use FPS by nearly four-fold.

**Table 5:** Odds of the training to increase parents’ knowledge and attitudes on adolescent sexuality topics

Outcome variables	Post intervention		
	OR	95% CI	*p*-value
Knowledge of parents on sexual reproductive health of adolescents	16.199	4.41–59.48	<.0001
Attitudes of parents approval towards adolescents use of family planning services	3.717	1.53–9.06	0.039

Estimates adjusted for age, sex and marital status

## Discussion

### Parents’ knowledge about adolescents’ sexuality topics

Parent training about sexuality topics was aimed at training parents to be able to communicate with their adolescents. The more knowledgeable a parent is about sexuality topics, the more confident s/he feels about discussing such topics with her/his adolescent. Parents’ knowledge about sexuality issues is very important when they are faced with the need to discuss and educate children on sexuality. Ubaidur et al. [13] in their study reported a lack of knowledge among parents on basic aspects of adolescent reproductive and sexuality health issues in Bangladesh. The parents in the current study, however, had varied levels of knowledge of adolescent sexuality topics. For instance, with topics relating to biological development, the parents in both the intervention and the control groups had good knowledge about them at pre- intervention. This is based on the assumption that these topics centred on common physical features that a parent may notice on an adolescent as s/he develops. Thus it is not surprising that most parents in this study had good or very good knowledge about these topics. Other factors that may contribute to the high knowledge about sexual development as shown in this study may include parent’s age, education, socio-economic status and parity as demonstrated in a previous study among European American mothers [14]. Demographic characteristics reported in this study shows that the parents were adults (not adolescents) and hence were expected to have much knowledge on biological development topics as reported in a previous study that compared biological development knowledge among adult and adolescent mothers [14].

Several studies in other countries have demonstrated sexual risk-taking behaviours among adolescents [15–17]. However, parents’ knowledge about adolescent sexual risk protection is low as about a third of parents in this study had little or no knowledge about this topic. At pre-intervention as reported in a randomized controlled trial in Virginia, USA, parents significantly underestimated their adolescents’ sexual risk behaviours [15]. However, there was increased similarity of reports by adolescents and their parents about adolescents’ involvement in risk and protective behaviours after the intervention. Thus there was a significant increase in knowledge about sexual risk-taking behaviours and protection over baseline values as reported in the Virginia study and this is in agreement with the findings of this present study. The significant increase seen in the intervention group may have been due to the importance parents placed on this topic as their level of knowledge at pre-intervention was low. It is likely that, they made the effort to learn about these risk protection topics so that they could educate their adolescents better against risky sexual behaviours. Previous evidence from North America shows that parent-adolescent communication about risky sexual behaviours reduces such behaviours among adolescents [17, 18]. Among African American parents, knowledge about sexual risk protection and the frequency of parent –adolescent communication influenced female adolescent sexual risk-taking behaviours positively [17]. Hence increase in knowledge of sexual risk protection topics among parents in the intervention group may have some beneficial effects on adolescent sexual risk protection, provided parents are able to effectively communicate the knowledge gained to the adolescent in a conflict-free manner.

Even though the benefits of contraceptive use were acknowledged, most parents lacked knowledge on this topic as reported in an earlier study in the United States [19]. Most parents in the study did not have knowledge of topics on contraceptive use. Many factors could account for the lack of knowledge of topics on contraceptive use, the most probable being parents’ fear that their adolescents would engage in sexual activities if such topics are discussed with them. In a similar study elsewhere, timing of first discussion of sexual intercourse and contraceptive use contributed an additional variance in several sexual risky behaviours among adolescents [16]. The increase in knowledge on contraceptive use among parents as reported in this study after the intervention is in agreement with previous findings from a study in Virginia, USA [15]. Parents’ knowledge on contraceptive use may benefit parents as well as adolescents in making sexual risky decisions [20, 21]. Also, there is some evidence, although not consistent, that knowledge about condoms and contraception and their effectiveness is related to condom or contraceptive use [22]. For example, parents and adolescents may not use contraceptives consistently and correctly if they do not know it exists, how to obtain it, or how to use it properly: knowing all of these information may be necessary for its proper use.

Innumerable studies have shown that parents have significant influences on the sexual and reproductive health of their children [15, 21, 23]. Indeed, the notion in Ghana is that talking about sex and sexual activities is a taboo, for which reason parents have difficulties discussing anything related to sex with their adolescents. Thus, this study examined parental knowledge on experiential sexual topics. Experiential sexual topics or issues were defined as experience of heterosexual or homosexual feelings. About half of the parents had little or no knowledge about whether their adolescent child had ever experienced homosexuality and/or had ever experienced sexual feelings. Many adolescent, parent, and family level factors may predict the accuracy of parent’s knowledge on experiential sexual topics. These factors undermining experiential sexual knowledge, as well as causes of tensions between adolescents and parents, needs to be elucidated and curtailed through healthy sex talks between parents and their adolescents.

In real life, knowledge provides a basis for human action but what people know does not necessarily affect what they do. This study revealed that about 20% of parents had little or no knowledge about sexuality topics. In a related study in Bangladesh, it was revealed that 65% of parents expressed their lack of knowledge about adolescent reproductive health issues and wanted to know more about the subject [24]. Another study in rural Tanzania showed that parents were limited as to what they could discuss with their adolescents about sexual and reproductive health issues because of lack of the appropriate knowledge, as well as cultural norms that prevented interaction between the opposite sexes [25]. Differences in religious and cultural norms may account for the differences in knowledge about sexuality topics from country to country. For example in Dakar, Senegal, religious leaders believed that parents should discuss reproductive health issues openly with their children, but parents lack the knowledge to do so with confidence [26]. On the contrary, talking about sex and sexual activities with adolescents in Ghana is perceived as a cultural taboo. A number of studies in the United States have suggested that Latino parents find difficulty in talking about the technical aspects of sexuality, including contraceptives and birth control because they require specialized knowledge [27, 28]. This may be due to the fact that many Latino parents lack the knowledge to discuss such topics or may think that talking about contraception with adolescents may encourage adolescent sexual activity. In agreement with the findings of this study, European American mothers demonstrated a fair but less than complete basic parental knowledge about sexuality [14]. This study further indicated that mother’s age and education independently contributed to their knowledge [14].

Three months after the sexuality health education training programme, parents’ knowledge significantly increased, with a higher proportion of parents in the intervention group showing an increase in their knowledge on sexuality topics as compared to their colleagues in the control group. In a review of the impact of programmes on knowledge about sexuality topics, it was concluded that majority of the programmes increased knowledge in one or more areas and this is consistent with the findings of this study [21]. This means that educating parents about sexuality has the potential to increase their knowledge, and this will enable them to talk to their adolescents about sexuality. On the other hand, even though knowledge may provide a foundation, it may not necessarily assure parental ability to talk to their adolescents about sexuality topics.

### Parents’ attitudes towards allowing adolescents to use family planning services

According to Kirby, knowledge is important because it provides the foundation for many values, attitudes, perceptions of norms, skills and ultimately for behaviour [21]. Hence parental knowledge and attitudes towards adolescent’s sexuality could either encourage or discourage adolescents from living healthy sexual lives. At pre-intervention, about 30% of the parents in the intervention and control groups indicated that they would not approve of their adolescents to use FPS. This finding is in agreement with an earlier study in Lesotho, which indicated that some parents, especially fathers, maintained that unmarried adolescents were not supposed to plan a family, therefore they should not be provided with reproductive health services [29]. After the training, there was a significant increase in the proportion of parents in the intervention group who reported that they would approve of their adolescents to access FPS. In keeping with previous studies, it was reported that majority of educational programmes do significantly improve attitudes about sex and condom/contraceptive use [21]. The training had provided the parents with enough information on contraceptive and condom use, thus they saw the need for adolescents to access such services.

As a measure of impact, the difference-in-difference estimates for parents’ attitudes towards allowing adolescents to use FPS was significant. This is an indication that educating parents about sexuality improved their attitudes towards allowing adolescents to use FPS. Adolescents’ access to FPS is not meant simply for contraceptive services alone, but also to enable them to seek information about their sexuality. Similarly, sexually active adolescents need to protect themselves against unprotected sex by the use of contraceptives like condoms, which they can access at family panning centres. It is important to note that parents who have “positive attitudes” towards allowing adolescents to use FPS are more likely to talk to their adolescents about it. If parents have “negative attitudes” towards it, they are less likely to talk about it or allow their adolescents to use it. Some proportion of parents were still ambivalent about adolescent use of FPS even after the intervention. This is in agreement with theories of attitudes change which indicate that people can be uncertain of how they feel about something even after continuous education [21, 30].

### Limitations

One limitation of this study is that, it was conducted in an urban setting, hence the findings cannot be generalized to parents in rural settings. Another possible limitation is that, the study did not survey the adolescents themselves to determine the effect of the programme on communication with parents about sexuality from the adolescent perspective. This limitation will be addressed in subsequent manuscripts from the project. Also, the relatively short period of the parent sex education programme may be a source of concern. Nonetheless, it demonstrates that educating parents on sexuality topics for a relatively short period of time can positively impact parent knowledge and attitudes about adolescent sexuality.

## Conclusion

A one month parents training on adolescent sexuality significantly increased parents’ knowledge about sexuality topics and positive attitudes towards allowing adolescents to use family planning services (3 months after the training). Scaling-up this parent training programme on adolescent sexuality by community-based organizations such as Planned Parenthood Association of Ghana (PPAG) and church groups may have some beneficial effects on parent-adolescent communication about sexuality and hence better adolescent reproductive health.

## Abbreviations

DID: Difference in difference model
FPS: Family Planning Services
SPSS: Statistical Package for the Social Sciences
